
JOURNAL INFORMATION
==============================
NLM Title Abbreviation: Int J Technol Assess Health Care
Journal ID: Int J Technol Assess Health Care
NLM Journal ID: 8508113
Journal ID: THC

International Journal of Technology Assessment in Health Care

ISSN: 0266-4623
EISSN: 1471-6348
Publisher: Cambridge University Press

ARTICLE INFORMATION
==============================
PMCID: PMC11719197
DOI: 10.1017/S0266462324004604
Article ID: S0266462324004604
Article version: 1
Subjects: Acknowledgements

Acknowledgements

Published online by Cambridge University Press

Publication date: 2024 Dec
Electronic publication date: 2025 Jan 7
Volume: 40
Issue: Suppl 1
Issue title: Abstracts from the HTAi 2024 Meeting in Seville, Spain
First page: i
Last page: i
Copyright: © The Author(s) 2024
Copyright year: 2024
Copyright holder: The Author(s)
License: This is an Open Access article, distributed under the terms of the Creative Commons Attribution licence (https://creativecommons.org/licenses/by/4.0/), which permits unrestricted re-use, distribution, and reproduction in any medium, provided the original work is properly cited.
License URL: https://creativecommons.org/licenses/by/4.0/

Page count: 1

==============================
Acknowledgements

The HTAi Supplement 2024 showcases abstracts from the HTAi meeting in Seville (15–19 June). Thanks to Ann Scott and Jo Vabolis for editing this edition and for the support of HTAi staff.

